# Clinical evaluation of a small implantable cardiac monitor with a long sensing vector

**DOI:** 10.1111/pace.13728

**Published:** 2019-06-05

**Authors:** Christopher Piorkowski, Mathias Busch, Georg Nölker, Jörn Schmitt, Franz Xaver Roithinger, Glenn Young, Miloš Táborský, Gundula Herrmann, Dietmar Schmitz

**Affiliations:** ^1^ Department of Invasive Electrophysiology Heart Center Dresden Dresden Germany; ^2^ Department of Internal Medicine B Greifswald University Hospital Greifswald Germany; ^3^ Clinic for Cardiology, Herz‐ und Diabeteszentrum NRW Ruhr‐Universität Bochum Bad Oeynhausen Germany; ^4^ Department of Cardiology University Hospital Giessen Giessen Germany; ^5^ Department of Inner Medicine Landesklinikum Mödling Mödling Austria; ^6^ Department of Cardiology St. Andrew's Hospital Adelaide South Australia Australia; ^7^ Department of Internal Medicine—Cardiology University Hospital Olomouc Olomouc Czech Republic; ^8^ Center of Clinical Research Biotronik SE & Co. KG Berlin Germany; ^9^ Clinic for Cardiology and Angiology St. Elisabeth Hospital Essen Essen Germany

**Keywords:** arrhythmia detection, atrial fibrillation, implantable cardiac monitor, remote monitoring, syncope

## Abstract

**Introduction:**

We conducted this study to show the safety and efficacy of a new implantable cardiac monitor (ICM), the BioMonitor 2 (Biotronik SE & Co. KG; Berlin, Germany), and to describe the arrhythmia detection performance.

**Methods:**

The BioMonitor 2 has an extended sensing vector and is implanted close to the heart. It can transmit up to six subcutaneous electrocardiogram strips by Home Monitoring each day. We enrolled 92 patients with a standard device indication for an ICM in a single‐arm, multicenter prospective trial. Patients were followed for 3 months, and 48‐h Holter recordings were used to evaluate the arrhythmia detection performance.

**Results:**

One patient withdrew consent and in one patient, the implantation failed. Two study device‐related serious adverse events were reported, satisfying the primary safety hypothesis. Implantations took 7.4 ± 4.4 min from skin cut to suture. At 1 week, the R‐wave amplitude was 0.75 ± 0.53 mV. In the 82 patients with completed Holter recordings, all patients with arrhythmias were correctly identified. False positive detections of arrhythmia were mostly irregular rhythms wrongly detected as atrial fibrillation (episode‐based positive predictive value 72.5%). Daily Home Monitoring transmission was 94.9% successful.

**Conclusion:**

Safety and efficacy of the new device has been demonstrated. The detected R‐wave amplitudes are large, leading to a low level of inappropriate detections due to over‐ or undersensing.

## 1 INTRODUCTION

Implantable cardiac monitors (ICMs) are used to detect infrequent cardiac rhythm disturbances. Capable of monitoring for a period of several years, ICMs can demonstrate correlation between symptoms and arrhythmia, guide medical therapy for atrial fibrillation (AF), and help risk stratification in structural heart disease.^1^, ^2^, ^3^, ^4^, ^5^ Remote monitoring has overcome the problem of limited ICM storage capacity (∼1 h of electrocardiogram [ECG]) and allows earlier detection of and response to clinically relevant arrhythmia.^2^, ^3^ Further device miniaturization, extended automation and features, and more accurate detection algorithms could expand the field of application for ICMs.^3^

The novel BioMonitor 2 ICM (Biotronik SE & Co. KG, Berlin, Germany) is a successor device to the BioMonitor,^4^, ^5^ with a USB‐stick‐like shape, 60% reduced weight and volume, and twice longer sensing vector to increase R‐wave amplitudes and improve diagnostic accuracy. The objective of the prospective, multicenter, single‐arm, nonrandomized BIO|MASTER.BioMonitor 2 study was to evaluate safety and efficacy of the BioMonitor 2 and its dedicated insertion tools.

## 2 METHODS

### 2.1 Patients

Study patients had to be at least 18 years old; be able and willing to comply with study procedures, including remote monitoring surveillance; and fulfill any of the following: (1) having a standard indication for ICM such as unexplained syncope or other symptoms possibly caused by heart rhythm disturbances or being (2) currently planned for ICM‐guided therapy management of paroxysmal AF, (3) indicated for catheter ablation of persistent AF, or (4) ablated for persistent AF within 4 weeks before enrolment.

Patients were excluded if they had any cardiac rhythm management device implanted (e.g., pacemaker), had life expectancy <6 months, were pregnant or breast‐feeding or considering becoming pregnant during the study, or if they participated in another interventional clinical investigation.

All patients provided written informed consent. The study was done in compliance with good clinical practice guidelines and the Declaration of Helsinki, including approval of the study protocol by appropriate national and local ethics committees, and study registration with ClinicalTrials.gov, number NCT02565238.

### 2.2 Device studied

**Figure 1:**
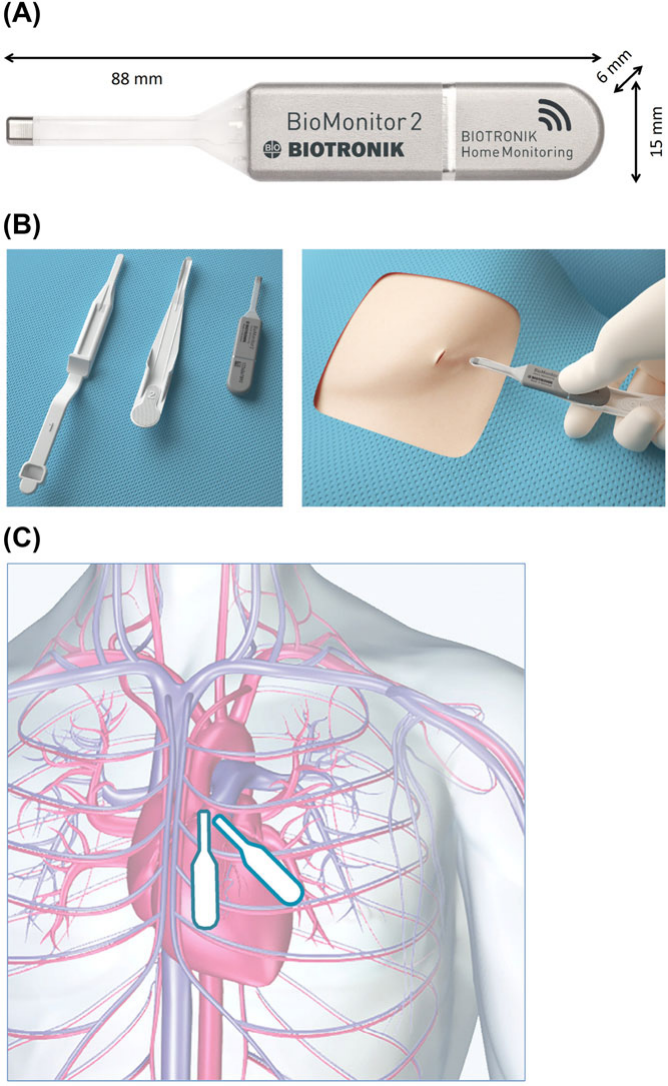
(A) BioMonitor 2 is composed of a combination of a rigid part (hermetically sealed titanium housing coated in silicone except for the electrode) and a flexible part (lead body composed of silicone, carrying titanium electrode and the antenna for Home Monitoring). (B) The fast insertion tool comprises a pocket tool to form device pocket and a lead support tool to facilitate insertion of the flexible lead. (C) Typical implant positions are parallel to the heart's long axis (diagonal) and straight (vertical) [Color figure can be viewed at http://wileyonlinelibrary.com]

The device has a volume of 5 cc and a weight of 10 g (Figure 1A). Attached to the 55‐mm × 15‐mm × 6‐mm rigid part is a flexible “antenna” of 33 mm, which can adapt to the shape of the body while extending the sensing vector to increase the signal amplitude. A dedicated fast insertion tool (FIT) set is provided to place the BioMonitor 2 into a subcutaneous pocket (Figure 1B). Typical ICM positions, diagonal and vertical, are illustrated in Figure 1C.

The sensing threshold is automatically adjusted based on the QRS amplitude and varies within the heart cycle. Based on a set of programmable criteria, five different types of heart rhythm disturbances can be detected automatically by the BioMonitor 2: asystole, bradycardia, AF, high ventricular rate, and sudden ventricular rate drop. AF is detected if a programmable cycle length variability is exceeded for longer than a programmable period. A subcutaneous ECG (sECG) snapshot lasting for 40 to 60 s is stored for up to 55 episodes of rhythm disturbances, before episodes are overwritten according to an algorithm that considers the clinical relevance. In addition, symptomatic patients can use the Remote Assistant^®^ device to initiate recording of a total of four sECG snapshots, each lasting for 7.5 to 10.0 min (0.5 min after triggering, the remainder prior to trigger).

The integrated Biotronik Home Monitoring^®^ technology^6^, ^7^ (in further text “Home Monitoring”) allows automated daily transmission of the ICM memory data including up to six sECG snapshots. Clinicians can view transmitted data on a secure, dedicated website regularly or after receiving configurable alert notifications.

The BioMonitor 2 integrates an active noise detection algorithm.^4^ If it detects a very high signal rate (>600/min), the arrhythmia detection algorithms are temporarily suspended to avoid false episode detections. The device records the percentage of the time spent in the noise mode as noise burden, allowing the quantification of sensing difficulties.

### 2.3 Study protocol

During ICM implantation, the implanters evaluated handling characteristics of the FIT set, separately for the pocket tool and the lead support tool.

Most devices were programmed to standard settings: asystole duration ≥3 s; bradycardia rate ≤40 beats/min for at least 10 s; high ventricular rate ≥180 beats/min for at least 16 beats; for AF, R‐R variability ≥12.5%, with confirmation time of 6 min; and sudden rate drop OFF.

Patients were followed for 3 months after ICM insertion. At the 1‐week and 3‐month follow‐ups, R‐wave amplitudes were measured, noise burden and other ICM data were retrieved, and adverse events and device deficiencies were assessed.

A continuous 48‐h Holter‐ECG obtained between the 1‐week and 3‐month follow‐ups was used to validate the corresponding ICM detections. Adjudication was done by one of the authors who did not enroll study patients (DS). Characteristics of binary classification, such as positive predictive value (PPV), negative predictive value, sensitivity, and specificity, were calculated for different arrhythmia types using episode‐based and patient‐based approaches, and for AF, also a duration‐based approach (see Appendix A).

### 2.4 Study endpoints

The primary endpoint was freedom from serious adverse device effects (SADEs) related to the BioMonitor 2 or FIT. The primary hypothesis was that the proportion of patients without SADE would be >90% from the beginning of implantation to study termination. The secondary hypothesis was that the mean R‐wave amplitude at the 1‐week follow‐up would be greater than 0.3 mV, the historical result for the predecessor device.^4^ Additional data of interest were handling characteristics of the FIT set, overall sensing behavior including noise burden, accuracy of arrhythmia detection by the ICM verified by Holter‐ECG findings, Home Monitoring transmission performance, and adverse events.

### 2.5 Statistical methods

The sample size was calculated using the POWER procedure of SAS version 9.4 (SAS Institute, Cary, NC, USA). For a significance level of *α* = .05, a statistical power of 1 – *β* = .9, binomial proportion of 0.98, and proportion of 0.9 (test limit), a sample size of 85 patients was required. Assuming a drop‐out rate of 2%, 87 patients had to be enrolled. It was thereafter decided that 30 patients should have an inclusion criterion not involving AF and 57 patients should have an AF‐related inclusion criterion to collect data on AF detection.

All endpoints were analyzed per protocol. The primary hypothesis was assessed by an exact binomial test. For continuous variables, descriptive statistics (mean ± standard deviation, median, and interquartile range [IQR]) were calculated and compared using the two‐sided Wilcoxon signed‐rank test. Nominal and ordinal variables are presented as absolute and relative frequencies. A *P*‐value of <.05 was considered statistically significant. All calculations were carried out using SAS version 9.4.

## 3 RESULTS

Between September 2015 and July 2016, 92 patients were enrolled at 13 investigational sites in Germany (10 sites), Australia (one), Austria (one), and Czech Republic (one) (see Appendix B). Table 1 shows patient characteristics. One patient withdrew informed consent before implantation. The insertion of the BioMonitor 2 was successful in 90 of 91 patients (98.9%). Insertion failure in one patient was caused by bent pocket tool.

**Table 1:** Baseline characteristics of enrolled patients

Parameter	Value N = 92
Age (years)	63 ± 13
Female gender	33 (36%)
History of atrial fibrillation	62 (67%)
Paroxysmal (self‐terminating within 48 hours)	44 (71% of 62)
Persistent (>7 days or requiring cardioversion)	16 (26%)
Permanent	2 (3%)
Main indication for insertable cardiac monitor	
Symptomatic or asymptomatic atrial fibrillation	44 (48%)
Cryptogenic stroke	15 (16%)
Syncope or presyncope	33 (36%)
Heart failure status	
No history of heart failure	63 (68%)
NYHA class I	14 (15%)
NYHA class II	9 (10%)
NYHA class III	6 (7%)
Comorbidities	
Hypertension	58 (63%)
Coronary artery disease	16 (17%)
Valvular heart disease	9 (10%)
Stroke	15 (16%)
Transient ischemic attack	3 (3%)
Thyroidism	7 (8%)
Chronic obstructive pulmonary disease	4 (4%)
Diabetes mellitus	13 (14%)
Renal insufficiency	11 (12%)

Data are mean ± standard deviation or number (percent).

NYHA = New York Heart Association.

Of the 90 patients with ICM, 84 terminated the study regularly (93.3%) and six prematurely (6.7%). The reason for premature termination were pacemaker implantation (N = 3; asystole or intermittent complete atrioventricular block detected by ICM), loss to follow‐up (N = 2), or withdrawal of consent (N = 1).

### 3.1 Primary hypothesis (safety)

The primary endpoint was evaluated in 91 patients undergoing ICM insertion. Two SADEs were reported, both being the risk of erosion resulting in ICM explantation. The SADE‐free rate was 97.8% (89/91; 95% confidence interval, 92.3‐99.7%). Because this is significantly higher than 90% (*P* = .004), the primary hypothesis is met.

### 3.2 Secondary hypothesis (R‐wave amplitude)

The R‐wave measurements at 1 week were available in 79 patients. The mean R‐wave amplitude of 0.75 ± 0.53 mV (median, 0.63; IQR, 0.41‐0.97) was significantly higher than the historical result of 0.30 mV for the BioMonitor^4^ (*P* < .001).

### 3.3 Handling characteristics of the insertion tool

In the 90 patients with successfully inserted ICM, the time from first skin cut to last suture was 7.4 ± 4.4 min (median, 6.1; IQR, 4.4‐9.3). One‐third of this time was spent on ICM positioning (mean, 2.8 ± 2.9 min; median, 1.8; IQR, 1.2‐3.0).

**Figure 2:**
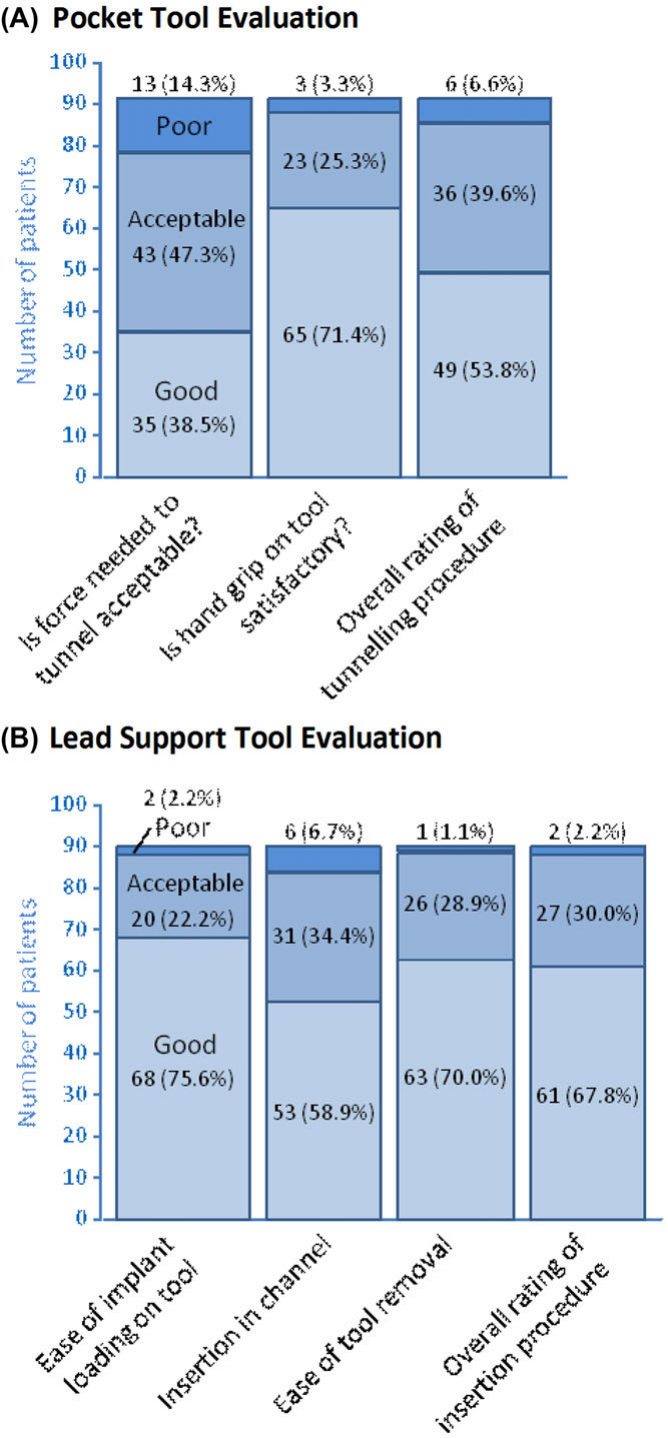
(A) The implanters’ evaluation of the tunneling procedure with the pocket tool in 91 patients. (B) The lead support tool evaluation in 90 patients. In all seven bars, the color scheme is the same (good/acceptable/poor in the upward direction) [Color figure can be viewed at http://wileyonlinelibrary.com]

The mean incision length was 16.4 ± 4.1 mm (median, 15.0; IQR, 15.0‐20.0). All devices were implanted subcutaneously, without the use of sutures to fixate the device. The force needed to tunnel and prepare the pocket was rated good or acceptable in 85.7% and poor in 14.3% of patients due to insufficient sharpness of the pocket tool, requiring substantial force on the tool or use of scissors. When hand grip evaluation is added, the overall rating of the tunneling procedure was good or acceptable in 93.4% and poor in 6.6% of patients (Figure 2A). The subsequent ICM insertion procedure was rated good or acceptable in 97.8% and poor in 2.2% of patients (Figure 2B).

The ICM was placed in a diagonal position in 63.3% (N = 57) and in a vertical position in 34.4% (N = 31) of patients (Figure 1C). In the remaining two patients, a 45° parasternal position was used (N = 1) or the position was not reported (N = 1).

### 3.4 Overall sensing behavior

There was no significant difference in the mean R‐wave amplitude at ICM insertion (0.81 ± 0.46 mV; median, 0.70; IQR, 0.45‐1.00), at the 1‐week follow‐up (reported above as secondary hypothesis), and at the 3‐month visit (0.73 ± 0.48 mV; median, 0.59; IQR, 0.40‐0.97). All measurements taken together, the diagonal ICM position tended to show a higher R‐wave amplitude (0.81 ± 0.51 mV) than the vertical position (0.69 ± 0.46 mV; *P* = .06).

The noise burden measured by ICMs from the 1‐week to the 3‐month follow‐up was 3.4 ± 7.5% (median, 1.0; IQR, 0.0‐3.0). The diagonal position was less susceptible to noise (2.3 ± 5.3%) than the vertical position (4.9 ± 9.9%; *P* = .01).

### 3.5 Accuracy of arrhythmia detection

Of the 90 patients with ICM, three refused Holter monitoring, one had the ICM explanted, one withdrew consent before Holter monitoring, and three had incomplete ICM memory export after Holter monitoring. The remaining 82 patients had usable Holter‐ECG (mean length, 47.7 ± 3.1 h) and the corresponding ICM data.

All 174 episodes of asystole, bradycardia, sudden rate drop, and high ventricular rate seen in Holter‐ECG were detected by the ICM (100% sensitivity; Table 2). Furthermore, of 98 AF episodes (≥6 min) in 15 patients, 95 were detected (96.9% sensitivity). Altogether, 269 of 272 arrhythmia episodes of all types were detected by the ICM (98.9% sensitivity). On the other hand, 46 cases of false positive detections reduced the overall PPV to 85.4% (Table 2). Most of them were AF detections; false detections because of undersensing were rare (three patients had false detections of bradycardia or asystole).

**Table 2:** Arrhythmia episodes

	Total	Asystole	Bradycardia	SRD	HVR	AF
Number of episodes in Holter‐ECG	272	3	105	25	41	98
Number of episodes detected by ICM	315	9	108	26	41	131
True positive (TP) episodes	269	3	105	25	41	95
False positive (FP) episodes	46	6^a^	3^a^	1^a^	0	36^b^
False negative (FN) episodes	3	0	0	0	0	3^c^
Sensitivity = TP/(TP+FN)	98.9%	100%	100%	100%	100%	96.9%
Positive predictive value = TP/(TP+FP)	85.4%	33.3%	97.2%	96.2%	100%	72.5%

*Note*. The analysis included 82 patients with adjudicated Holter findings and ICM data. For details on evaluation methodology, see Appendix 1.

a Due to undersensing.

b Due to unstable rhythm caused by ventricular extrasystoles or atrial ectopic activity (mimicking R‐R interval irregularity typical for AF), except for one episode of P‐wave oversensing.

c Three episodes of AF were evident in the Holter‐ECG but were not detected by the ICM in a single patient having AF alternating with periods of atrial flutter, resulting in too many pseudo‐regular intervals.

Abbreviations: AF = atrial fibrillation; ECG = electrocardiogram; ICM = insertable cardiac monitor; HVR = high ventricular rate; SRD = sudden ventricular rate drop.

Patient‐based sensitivity was 100%, because all patients with arrhythmia were properly identified by the ICM for all rhythms (Table 3). Reduced by false positive cases, patient‐based specificity ranged from 88.1% for AF to 97.5‐100% for other arrhythmia.

**Table 3:** Patient‐based arrhythmia detection results

	Asystole	Bradycardia	SRD	HVR	AF
Patients with episodes in Holter‐ECG	2	7	2	3	15
Patients with episodes detected by ICM	4	8	3	3	23
True positive (TP) patients	2	7	2	3	15
False positive (FP) patients	2	1	1	0	8
False negative (FN) patients	0	0	0	0	0
True negative (TN) patients	78	74	79	79	59
Sensitivity = TP/(TP+FN)	100%	100%	100%	100%	100%
Specificity = TN/(TN+FP)	97.5%	98.7%	98.8%	100%	88.1%
Positive predictive value = TP/(TP+FP)	50.0%	87.5%	66.7%	100%	65.2%
Negative predictive value = TN/(TN+FN)	100%	100%	100%	100%	100%
Accuracy = (TP+TN)/(TP+TN+FP+FN)	97.6%	98.8%	98.8%	100%	90.2%

*Note*. The analysis included 82 patients with adjudicated Holter findings and ICM data (see Appendix 1 for details).

Abbreviations: AF = atrial fibrillation; ECG = electrocardiogram; ICM = insertable cardiac monitor; HVR = high ventricular rate; SRD = sudden ventricular rate drop.

The adjudicated cumulative duration of AF was 401 h out of 3913 h of pooled Holter‐ECG recordings for all patients (10.3% of time in AF). Of these 401 h, 376 h were detected as AF by the ICM (93.6% duration‐based sensitivity). Together with 27 h of false AF detection, this resulted in an AF duration‐based specificity of 99.2%.

### 3.6 Home monitoring transmission performance

The ratio of the number of days with a transmitted message and the number of days in the transmission period (day of first transmitted message to day of last transmitted message of each patient) was 94.9% in pooled data for all patients.

### 3.7 Adverse events

Ten device‐related adverse events were reported in eight patients. Of the two serious events that were evaluated as primary endpoints, one was connected to an infection. Eight adverse device effects were nonserious, including acute (N = 2) and late (N = 2) pain or nausea, hematoma (N = 2), device migration (N = 1), and infection (N = 1).

## 4 DISCUSSION

The new ICM (BioMonitor 2) was developed to facilitate minimally invasive surgical procedure by reduced device size and to improve R‐wave amplitudes and arrhythmia detection by a longer sensing vector. The present study demonstrated the safety and efficacy of the BioMonitor 2 and its FIT set.

The safety was shown by an SADE‐free rate of 97.8%, which is significantly higher than the hypothesized 90%. The efficacy was indicated by the increased R‐wave amplitude, reliable arrhythmia detection, and good handling characteristics of the FIT set.

### 4.1 Sensing performance

The mean R‐wave amplitude of 0.75 mV for the BioMonitor 2 is markedly larger than 0.3 mV for the predecessor device,^4^ owing to the longer sensing vector and the implantation closer to the heart. It compares favorably with the other devices on the market (eg, 25% larger than Medtronic LINQ^TM^)^8^, ^9^ and leads to an improved noise burden, which has been reduced considerably, from median 4.0% (BioMonitor)^4^ to 1.0% (BioMonitor 2) at the 3‐month follow‐up. This figure has not been reported for other devices; translated into practical terms, it means that the patient's rhythm is monitored for 23 h and 45 min per day.

The diagonal BioMonitor 2 position tended to be associated with larger R‐waves and less noise than the vertical position. Although this finding should be taken cautiously because the allocation to the two positions was not randomized, it is plausible because a diagonal position is parallel to the heart's long axis.

### 4.2 Arrhythmia detection accuracy

Compared to its predecessor, the BioMonitor 2 showed improved sensitivity and specificity of arrhythmia detection. The BioMonitor 2 exhibited 100% episode‐based and patient‐based sensitivity for asystole, bradycardia, sudden rate drop, and high ventricular rate. For AF detection, patient‐based sensitivity was 100% and episode‐based sensitivity was 96.9%. This represents an improved detection efficacy compared to the BioMonitor (74.7%^5^ and 91.9%^4^).

Also the PPV was improved with the BioMonitor 2, especially for high ventricular rate (from 17%^4^ to 100%), bradycardia (from 41%^4^ to 97.2%), AF (from 59%^4^ to 72.5%), and remained unchanged for asystole (31%^4^ and 33.3%). The clear improvements in bradycardia and in high ventricular rate detection can be plausibly attributed to less undersensing due to higher R‐wave amplitudes, and to less oversensing because the higher amplitudes required a lower amplifier gain. Inappropriate detections especially for undersensing may be an issue in clinical practice. One study with the Reveal LINQ found them in 29% of the patients during long‐term use.^9^ We identified three of 82 (3.7%) patients with such episodes in our short‐term observation.

False‐positive detections, which are also reduced but not eradicated with the BioMonitor 2, will rarely lead to unnecessary treatments because ICM misclassifications can be largely overcome by manual analysis of the corresponding sECG snapshots by the physician.^5^ Even if the snapshots are overwritten due to ICM memory limitation, they may be available in the remote monitoring system archive.^5^ However, inappropriate detections remain an issue to be solved because of the workload connected to it. It should be kept in mind that the devices in this study were mostly set to standard programming, which can be optimized depending on the patient's indication.

The majority of inappropriate detections were caused by irregular rhythms detected as AF. One possibility to improve the performance of AF detection is to extend the minimum duration for an AF episode to be detected.^10^ Unfortunately, the fact that our device programming was not standardized and our limited sample size limits our ability to report on differences in performance depending on detection criteria.

Detection accuracy of the BioMonitor 2 does not differ meaningfully from that demonstrated in ICMs from other vendors. For example, in the Reveal XT Performance Trial (XPECT), the detection algorithm of the Reveal XT ICM (Medtronic, Minneapolis, MN, USA) identified AF patients with a sensitivity of 96.1% and specificity of 85.4% (R‐R interval variation analysis),^11^ compared with 100% sensitivity and 88.1% specificity for the patient‐based approach in our study. For the St. Jude Medical Confirm^TM^ device (St. Jude Medical, St. Paul, MN, USA), 100% sensitivity and 85.7% specificity have been reported in a similar approach.^12^ The successor device to Reveal XT, Reveal LINQ, using both R‐R interval variation and a new P‐wave recognition algorithm for AF detection, demonstrated an improved sensitivity of 97.4% and specificity of 97.0% for patient‐based analysis of AF.^13^ In the duration‐based approach, Reveal LINQ showed 98.4% sensitivity and 99.5% specificity,^13^ compared with 93.6% and 99.2% for the BioMonitor 2, respectively (Table 4). However, real‐world performance of devices is often worse than in controlled trials and depends on the AF incidence in the monitored population, the programmed sensitivity of AF algorithm, and the duration of detected AF episodes.^10^

**Table 4:** AF duration‐based analysis

	Total
Cumulative duration of Holter‐ECG recordings (h)	3913
Duration of AF in Holter findings (h)	401
AF‐free time in Holter findings (h)	3512
AF detected by ICM (h)	402
True positive (TP) time period (h)	376
False positive (FP) time period (h)	27
False negative (FN) time period (h)	25
True negative (TN) time period (h)	3485
Sensitivity = TP/(TP+FN)	93.6%
Specificity = TN/(TN+FP)	99.2%
Positive predictive value = TP/(TP+FP)	93.4%
Negative predictive value = TN/(TN+FN)	99.3%
Accuracy = (TP+TN)/(TP+TN+FP+FN)	98.7%

*Note*. The analysis included 82 patients with adjudicated Holter findings and ICM data (see Appendix 1 for details).

Abbreviations: AF = atrial fibrillation; ECG = electrocardiogram; ICM = insertable cardiac monitor.

For other rhythm disturbances (asystole and bradycardia), long‐term data from the BioMonitor 2 are needed to decide if there are major differences in detection accuracy between Reveal LINQ^9^, ^14^ and the BioMonitor 2. Detection of high ventricular rate cannot be compared because of different arrhythmia definitions across devices,^15^ whereas sudden rate drop is a unique feature of the BioMonitor 2.

### 4.3 Further results

The rating of the FIT set by BioMonitor 2 implanters was good or acceptable in the vast majority of patients, with the implantation procedure lasting for a median of 6.1 min (first cut to last suture). The ICM miniaturization trend continues and allows safe, minimally invasive surgical procedures.^2^, ^16^, ^17^ Although the Reveal LINQ (weight 2.5 g, volume 1.2 cm^3^, and insertion opening 8 mm) is smaller than the BioMonitor 2, this is at the expense of reduced device longevity (nominal battery life expectancy for Reveal LINQ of 2.5‐3.0 years vs BioMonitor 2 4‐6 years).^5^ Longer battery life (e.g., longer rhythm monitoring) may be less relevant for classical ICM indications than in novel attempts for risk management in patients with myocardial infarction (NCT02594488 and NCT02341534).

The success of daily remote monitoring transmission of 95% compares favorably with published figures (80%).^8^ A very reliable remote monitoring system may be especially important in ICM patients who are otherwise well suited for pure remote follow‐up.

### 4.4 Study limitations

The study had several limitations. First, it did not assess the long‐term sensing performance, but it may be assumed that sensing remains stable after wound healing that is largely completed within 3 months of ICM insertion. Second, the estimation of arrhythmia detection accuracy was limited by the short observation period under Holter monitoring (48 h). Additionally, only AF episodes ≥6 min in duration were included in the analysis; therefore, the study results are not applicable to episodes that are shorter than 6 min. Further, all comparisons to results of other devices are limited by the historical nature of the comparison data and by relatively low numbers of cases. Finally, due to the limited follow‐up of 3 months, we are not able to present data on device longevity.

## 5 CONCLUSION

The BioMonitor 2 is a new miniaturized ICM. With an implantation close to the V2 and V3 positions of the standard ECG and a very long sensing vector, the sensing is probably close to the theoretical optimum. Incorrect arrhythmia detections are typically irregular rhythms mimicking AF and can be overcome only by further algorithmic improvements. The device's size and the minimal invasiveness of insertion allow the use in established and in possible new fields of indication, such as AF management or assessment of the arrhythmia related risk in larger populations.

## CONFLICT OF INTERESTS

C.P. reports research support and speakers honoraria from Biotronik. M.B. is conducting research sponsored by Abbott, Biotronik and Medtronic and has received speakers honoraria from Boston Scientific and Medtronic. G.N. reports speaker honoraria from Medtronic. J.S. has received research support, consultancy fees and speakers honoraria from Biotronik. G.H. is a Biotronik employee. The other authors report no conflict of interest.

## APPENDIX A METHODS TO EVALUATE ACCURACY OF ARRHYTHMIA DETECTION

### A.1

The performance of any diagnostic test (“does the patient have a given condition?“) can be described with the method of binary classification. Four cases are possible:

True positive (TP): The diagnostic test shows that the patient has the condition in question and he/she does have it.
False positive (FP): The diagnostic test shows that the patient has the condition in question but he/she does not have it.
True negative (TN): The diagnostic test shows that the patient does not have the condition in question and he/she does not have it.
False negative (FN): The diagnostic test shows that the patient does not have the condition in question but he/she has it.

From the TP, FP, TN, and FN, the following performance characteristics can be defined:

Sensitivity: TP/(TP + FN)
Specificity: TN/(TN + FP)
Positive predictive value (PPV): TP/(TP + FP)
Negative predictive value (NPV): TN/(TN + FN)
Accuracy: (TP + TN)/(TP + TN + FP + FN).

In the present study, this matrix of performance characteristics was calculated for the episode‐based and the patient‐based arrhythmia detection accuracy, as explained below. For AF, also duration‐based analysis was made. All results were derived from the adjudicated 48‐h Holter‐ECG and BioMonitor 2 ICM findings.

### Episode‐based approach

In the episode‐based approach, all episodes are counted equally, irrespective of the patient who had the episode. The start time and the end time of an arrhythmia episode never match exactly in the BioMonitor 2 recordings and in the corresponding Holter‐ECG. The two ECG segments from different devices were therefore considered to represent a single episode if they overlapped temporarily. An episode detected by the BioMonitor 2 with the arrhythmia not visible between the start time and the end time in the Holter‐ECG was counted as a FP episode, while a short overlap was sufficient to make it a TP episode. FN episodes were those seen in the Holter‐ECG that had no overlapping episode detected by the BioMonitor 2.

A further condition for an episode to be considered as FN or not FN is that heart rhythm disturbances could be detected by the BioMonitor 2 only if they fulfill the programmable parameters defining the necessary duration (for asystole and AF) and rate (for bradycardia and high ventricular rate) criteria.

A patient with persistent AF in the Holter‐ECG and 10 detections of AF by the BioMonitor 2 in the same period would have 10 TP detections and no FN detection, although some periods of the ongoing AF episode have not been detected. This is because the single true episode has been detected at least once, and all 10 BioMonitor 2 episodes do overlap with true AF.

In contrast to TP, FP, and FN, the TN episodes cannot be defined and calculated meaningfully in the episode‐based approach. Therefore, only PPV and sensitivity can be determined for this approach, while NPV, specificity, and accuracy cannot be determined.

### Patient‐based approach

The patient‐based approach refers to an analysis that classifies patients as with or without a certain arrhythmia type occurring during the 48‐h Holter‐ECG recording, and with or without BioMonitor 2‐detected episodes of this type during this period. A patient with some FN and at least one TP detection of AF counts as TP patient, because he or she has been correctly identified as having AF.

All characteristics of binary classification, including TN, can be calculated, where “detection” refers to the BioMonitor 2 and “episodes” refer to the Holter monitoring:

Sensitivity: Patients with true detections (TP)/patients with any episodes (TP + FN)
Specificity: Patients with no detections & no episodes (TN)/patients with no episodes (TN + FP)
PPV: Patients with true detection (TP)/patients with any detection (TP + FP)
NPV: Patients with no detection and no episodes (TN) / patients with no detections (TN + FN)
Accuracy: (TP + TN)/(TP + TN + FP + FN).

### Duration‐based analysis in AF

The clinical investigation protocol required to synchronize the clocks of the BioMonitor 2 and the Holter device at the time of Holter start. The periods of AF as seen by the BioMonitor 2 and by Holter monitoring were aligned time wise; the timeline started and ended according to Holter. The AF episodes ongoing at Holter start were included. The following time periods were determined:

True positive (TP): Both BioMonitor 2 and Holter see AF.
False positive (FP): BioMonitor 2 sees AF but Holter does not see AF.
True negative (TN): Both BioMonitor 2 and Holter do not see AF.
False negative (FN): BioMonitor 2 does not see AF while Holter sees AF.

The AF timelines according to adjudicated Holter findings in 82 patients were determined as follows:

- In 67 patients without AF, the whole Holter timeline was “no AF.”
- In six patients with incessant AF, the whole Holter timeline was “AF.”
- Of nine patients with intermittent AF, raw Holter recordings were available at the time of this analysis in four patients.
  ○ In four patients with intermittent AF and raw Holter data, the Holter program was used to identify the onset and the end of AF episodes automatically. Episodes shorter than 6 min were discarded because they could not be detected by the ICM due to programmed AF confirmation time of 6 min.
  ○ In five patients with intermittent AF and no raw Holter data, the printout of the original analysis contained a graphical presentation of the periods in which the automatic analysis found AF. The start and the end times of these episodes were identified manually in the printouts.
    ■ In one patient, the Holter program did not recognize AF that was evident from the presence or absence of P‐waves and from the changing cycle lengths in the ECG. The decision of the adjudicator to classify this episode as AF overruled the automatic AF recognition of the Holter system.

The ICM‐based AF timelines were derived from the adjudicated list of episodes stored in the ICM.

## APPENDIX B INVESTIGATIONAL SITES AND NUMBER OF ENROLLED PATIENTS

### B.1

Klinikum der Ernst‐Moritz‐Arndt Universität Greifswald, Germany (N = 18);

Herz‐ und Diabeteszentrum Bad Oeynhausen, Germany (N = 17);

Universitätsklinikum Giessen, Germany (N = 10);

Landesklinikum Mödling, Austria (N = 8);

Herzzentrum Dresden, Germany (N = 8);

St. Andrew's Hospital, Adelaide, Australia (N = 7);

FN Olomouc, Czech Republic (N = 7);

Ev. Freikirchliches Krankenhaus Bernau und Herzzentrum Brandenburg, Germany (N = 4);

St.‐Marien‐Hospital GmbH Lünen, Germany (N = 3);

Cardiological Praxis Dr. med. Placke, Rostock, Germany (N = 3);

Klinikum Ludwigsburg, Germany (N = 3);

Universitätsklinikum Schleswig‐Holstein, Lübeck, Germany (N = 2);

DRK Krankenhaus Mölln/Ratzeburg gGmbH, Germany (N = 2).
